# Does Sex Matter in Obesity-Induced Periodontal Inflammation in the SS^LepR^ Mutant Rats?

**DOI:** 10.3390/dj13010014

**Published:** 2024-12-27

**Authors:** Abdulmohsin Alhashim, Kim Capehart, Jocelyn Tang, Karim M. Saad, Rafik Abdelsayed, Marion A. Cooley, Jan M. Williams, Ahmed A. Elmarakby

**Affiliations:** 1 Departments of General Dentistry and Oral Biology & Diagnostic Sciences, Dental College of Georgia, Augusta University, Augusta, GA 30912, USA; aalhashim@augusta.edu (A.A.); kcapehart@augusta.edu (K.C.); 2 Oral Biology & Diagnostic Sciences, Dental College of Georgia, Augusta University, Augusta, GA 30912, USA; jtang@augusta.edu (J.T.); ksaad@augusta.edu (K.M.S.); rabdelsa@augusta.edu (R.A.); mcooley@augusta.edu (M.A.C.); 3 Department of Pharmacology & Toxicology, Faculty of Pharmacy, Mansoura University, Mansoura 35516, Egypt; 4 Department of Pharmacology and Toxicology, University of Mississippi Medical Center, Jackson, MS 39216, USA; jmwilliams5@umc.edu

**Keywords:** obesity, periodontal inflammation, sex, insulin resistance, SS^LepR^ mutant rats

## Abstract

**Introduction:** The incidence of obesity has dramatically increased worldwide. Obesity has been shown to exacerbate the progression of periodontal disease. Studies suggest a sex difference in periodontitis, whereby males are more sensitive to periodontal inflammation compared to females. **Aim:** In the current study, it was hypothesized that obesity drives periodontal inflammation and bone loss in both sexes. **Methodology:** Utilizing leptin receptor mutant (SS^LepR^ mutant) rats as a genetic model of obesity, 11–12-week-old male and female lean Dahl salt-sensitive (SS) rats and obese SS^LepR^ mutant rats were used to investigate sex differences in obesity-induced periodontal inflammation. **Results:** Body weight, insulin, hemoglobin A1c and cholesterol levels were significantly elevated in the obese SS^LepR^ mutant strain vs. the lean SS strain within the same sex. Sex differences in body weight and plasma hemoglobin A1c were only observed in obese SS^LepR^ mutant rats, with males having significantly greater body weight and hemoglobin A1c vs. females. Plasma thiobarbituric acid reactive substances (TBARs) and monocyte chemoattractant protein-1 (MCP-1), markers of systemic oxidative stress and inflammation, respectively, were significantly elevated in obese SS^LepR^ mutant rats vs. lean SS rats, with no sex differences in these parameters in either rat strains. Although micro-CT analyses of the maxillary first molar alveolar bone from obese SS^LepR^ mutant rats revealed no evidence of bone loss and/or sex differences, immuno-histochemical analysis revealed significant elevations in periodontal IL-6 and decreases in IL-10 in obese SS^LepR^ mutant rats vs. lean SS rats, with no apparent sex differences in these parameters. **Conclusions:** Obesity increases systemic and periodontal inflammation, without evidence of bone loss or apparent sex differences in SS^LepR^ mutant rats.

## 1. Introduction

Periodontal disease, also called gum disease, usually starts with the infection and inflammation of soft tissues (gingivitis), and can advance to the inflammation of the supporting tissue of teeth (periodontitis) [1]. According to the Center for Disease Control (CDC), 47% of adults, aged 30 years or older, have periodontitis [2]. If left untreated, periodontitis can lead to progressive bone and teeth loss [3]. In addition, periodontitis is believed to increase the risk for vascular inflammation [3].

Studies have demonstrated gender differences in the incidence and progression of periodontal disease since the prevalence of periodontitis has been shown to be higher in men (57%) than women (39%) [4,5]. However, one mice model of periodontitis has shown conflicting data to the clinical findings, wherein female mice displayed significantly increased periodontal bone loss with elevations in pro-inflammatory cytokines’ expression and higher numbers of oral bacteria than males [6]. Many clinical factors contribute to the gender differences in periodontitis, such as smoking, poor oral hygiene and obesity. Since periodontitis is considered a chronic inflammatory disease, gender differences might interfere with the clinical signs and symptoms of the disease, as well as the response to therapeutic intervention. Thus, more work is needed to determine possible molecular mechanisms of gender differences in periodontal disease to optimize treatment options for both sexes.

The prevalence of obesity has increased recently, resulting in a higher risk for cardiovascular disease (CVD) [7,8]. Obesity is often associated with metabolic syndrome, which is characterized by insulin resistance, hypertension, hyperglycemia, and dyslipidemia [7,8]. Previous findings suggest that obesity increases the incidence of periodontal disease, where elevations in oxidative stress and inflammatory cytokine production are key elements in the progression of vascular injury [9]. Increases in weight are positively associated with vascular injury and periodontitis in both sexes [10,11]. Current treatments for obesity-induced vascular injury involve glycemic control via reducing body weight and sugar intake [12,13]. However, these approaches are modestly successful in delaying the progression of vascular injury [13,14]. Few studies have shown that obesity alone could drive gingival inflammation in rodents without the induction of periodontitis experimentally [15]. There is controversy in the literature regarding the relative susceptibility of males vs. females to obesity-induced vascular injury. To investigate possible gender differences in obesity-induced periodontal inflammation, obese SS leptin receptor mutant (SS^LepR^ mutant) rats were utilized as a genetic model of obesity in the current study. These rats were generated with a Dahl salt-sensitive genetic background using a Zinc-finger construct that caused a 16-base-pair frame shift deletion in the leptin receptor gene [16,17]. These rats have been shown to develop typical symptoms of metabolic syndrome (obesity, hyperlipidemia and insulin resistance) without elevations in blood pressure and hyperglycemia as early as 6 weeks of age [16,17].

## 2. Methods

A power analysis was initially performed, and it was estimated that n = 6 rats per group would have 80% power (α = 0.05) to detect a 50% difference in periodontal inflammation and injury between groups. Accordingly, experiments were conducted in 11–12-week-old male and female lean Dahl salt-sensitive (SS) and obese SS leptin receptor mutant (SS^LepR^ mutant) rats (n = 6–7 per group). Both strains were obtained from Dr. Jan William’s laboratory and were originally developed at the Medical College of Wisconsin utilizing Zinc-finger nuclease technology [17]. Both rat strains were maintained at the University of Mississippi Medical Center, approved by the American Association for the Accreditation of Laboratory Animal Care, and all protocols were approved by the University of Mississippi Medical Center. Rat genotypes were verified by the Molecular and Genomics Facility at the University of Mississippi Medical Center before starting the experiments. All rats were allowed free access to food and water throughout the study. Body weight was recorded before rats were anesthetized with 2% isoflurane to collect blood from the aorta. Blood glucose levels were monitored using a OneTouch Ultra glucometer (Life-Scan, Malvern, PA, USA). Blood was also used to assess hemoglobin A1c levels using A1cNow Self check (PTS Diagnostics, Whitestown, IN, USA). Plasma was isolated and used to assess insulin and cholesterol levels using commercially available kits from Millipore (Billeria, MA, USA) and Wako Diagnostics (Richmond, VA, USA), respectively. Plasma levels of thiobarbituric acid reactive substance (TBARs, Cayman chemicals, Ann Arbor, MI, USA) and monocyte-chemoattractant protein-1 (MCP-1, BD biosciences, Franklin Lakes, NJ, USA) were measured as markers of systemic oxidative stress and inflammation, respectively.

### 2.1. Histological Assessment of Periodontal Inflammation and Injury

Maxilla was isolated from all rat groups before being dissected, decalcified, embedded and sectioned as previously described [18]. Here, 5 µm-thick sections were obtained in the sagittal direction along the long axis of the teeth, and the sections were stained with hematoxylin and eosin (H&E). Additional maxilla sections were utilized for immune–histochemical assessments of myeloperoxidase, IL-6 and IL-10. These markers were selected based on a previous observation that myeloperoxidase and IL-6 were elevated in rodents with induced periodontal diseases, and could serve as inflammatory markers of periodontitis [19,20], whereas mice lacking IL-10 developed more alveolar bone loss with induced periodontal disease because IL-10 is an-inflammatory cytokine that limits immune response to a pathogen [21]. Briefly, paraffinized sections were dewaxed, hydrated, and immersed in antigen retrieval solution. Sections were treated with 1% hydrogen peroxide before overnight incubation with rabbit anti-myeloperoxidase polyclonal antibody (Thermo-Fisher, Waltham, MA, USA), rabbit polyclonal IL-6 antibody (Protein-tech, Rosemont, IL, USA) or rabbit polyclonal IL-10 antibody (Abclonal, Woburn, MA, USA). The slides were washed with PBS followed by incubation with anti-rabbit IgG HRP-secondary antibody (Vector laboratories, Newark, CA, USA) for 30 min, followed by the addition of substrate chromogen AEC (Vector laboratories, Newark, CA, USA), before the slides were counterstained using hematoxylin. Then, 8–10 images were taken for each slide at 200× magnification power. The numbers of myeloperoxidase-, IL-6- and IL-10-positive cells per mm^2^ were counted in each image blindly.

### 2.2. Micro-CT Analysis

The maxilla from all rat groups were scanned by a Bruker SkyScan 1272 micro-CT system (v2214, Bruker MicroCT, Kontich, Belgium) as described previously [22]. The degree of periodontitis was calculated by measuring bone loss from the cemento-enamel junction to the alveolar bone crest in the buccal side of the maxillary molar.

### 2.3. Statistical Analysis

All data are presented as means ± SEM. Statistical analysis was carried out utilizing GraphPad Prism 8.0.2 software (San Diego, CA, USA). The significance of the difference between the two rat strains and sex was determined using two-way ANOVA followed by Tukey’s post-hoc test for multiple comparisons. *p*-values of <0.05 were considered significantly different.

## 3. Results

### 3.1. Sex Differences in Obesity-Induced Changes in Metabolic Parameters

Body weight and the levels of hemoglobin A1c, glucose, cholesterol, and insulin were assessed in male and female lean Dahl salt-sensitive (SS) and obese SS leptin receptor (SS^LepR^) mutant rats at 11–12 weeks of age. As shown in Table 1, body weight, cholesterol and insulin levels were significantly elevated in the obese SS^LepR^ mutant strain compared with the lean SS rat strain, which findings are consistent with previous findings [17]. A sex difference in body weight was noted in both lean SS and obese SS^LepR^ mutant rats, whereby females had significantly lower body weights than males in both strains (Table 1). Obese SS^LepR^ mutant rats showed significant elevations in body weight compared to their age-matched lean SS rats (Table 1). There was a significant elevation in hemoglobin A1c levels in male obese SS^LepR^ mutant rats compared with lean SS rats regardless of their sex (Table 1). Hemoglobin A1c levels in male obese SS^LepR^ mutant rats were also significantly greater than in female obese SS^LepR^ mutant rats (Table 1), suggesting that male obese SS^LepR^ mutant rats might be more sensitive to obesity-induced cardio-metabolic symptoms than females. Similarly to in previous studies [16,17], there were no differences in blood glucose levels in SS and SS^LepR^ mutant (Table 1).

### 3.2. Obesity-Induced Elevations in Systemic Inflammation with No Apparent Sex Difference

**Figure 1:**
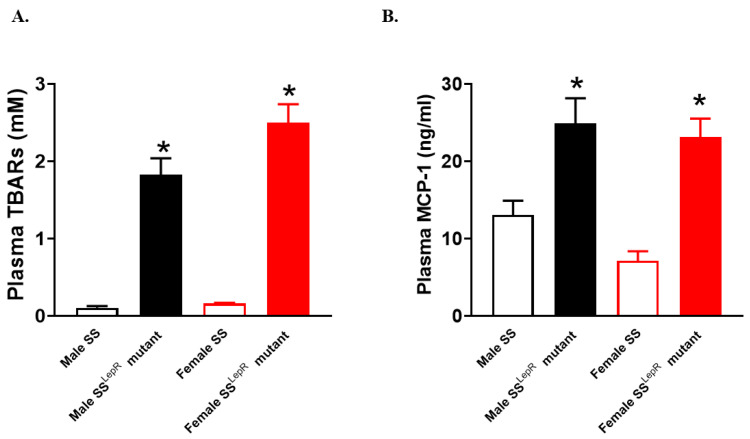
Obesity increased systemic markers of oxidative stress and inflammation in both sexes. Plasma TBARs level as a marker of oxidative stress (**A**) and plasma MCP-1 level as a marker of inflammation (**B**) in male and female lean Dahl salt-sensitive (SS) and obese SS leptin receptor mutant (SS^LepR^ mutant) rats. Values are presented as means ± SEM. * *p* < 0.05 vs. male or female Dahl SS rats (n = 6–7/group).

Since oxidative stress and inflammation are key factors affecting the progression in vascular injury in obesity [9], markers of systemic oxidative stress and inflammation were assessed in both rat strains (Figure 1). Plasma TBARs levels, a marker of oxidative stress, were significantly elevated in both male and female obese SS^LepR^ mutant rats vs. lean SS rats, with no apparent sex difference (Figure 1A). Similarly, the levels of plasma MCP-1, a marker of inflammation and a key chemokine in regulating monocyte/macrophage infiltration and migration [23], were also elevated in male and female obese SS^LepR^ mutant rats vs. lean SS rats, with no apparent sex difference (*p* ˂ 0.05, Figure 1B). These data suggest that obesity is associated with the elevation of both systemic oxidative stress and inflammation, which could be causative factors for vascular injury in both sexes.

### 3.3. Obesity Rats Did Not Show Evidence of Alveolar Bone Loss

**Figure 2:**
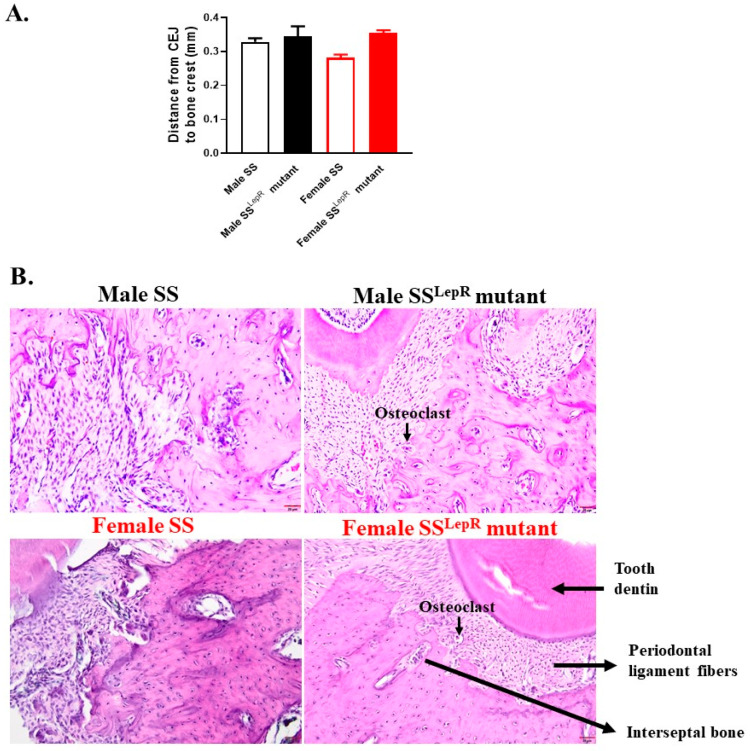
Obesity did not increase bone loss in both sexes. Degree of periodontitis was calculated by measuring bone loss from cemento-enamel junction (CEJ) to the alveolar bone crest in the buccal side of the maxillary first molar in male and female lean Dahl salt-sensitive (SS) and obese SS leptin receptor mutant (SS^LepR^ mutant) rats (**A**). Values are presented as mean ± SEM. (**B**) Representative images of maxilla sections from both male and female lean Dahl salt-sensitive (SS) and obese SS leptin receptor mutant (SS^LepR^ mutant) rats stained with hematoxylin and eosin (H&E). Images were captured at 200× magnification (n = 4/group).

To further determine if elevation in systemic oxidative stress and inflammation in our obese rat model could translate to periodontal inflammation, histopathological changes in periodontium in our two rat strains were assessed using maxillary sections stained with H&E (Figure 2). Cross-sections of periodontium interseptal bone showed the presence of more osteoclasts in SS^LepR^ mutant rats vs. SS, with no apparent sex differences (Figure 2A). The increase in osteoclast in obese animals was not associated with elevation in bone loss, since micro-CT analyses of the maxillary first molar from obese SS^LepR^ mutant rats revealed no evidence of significant alveolar bone loss and/or sex differences when compared to lean SS rats (Figure 2B).

### 3.4. Obesity-Induced Periodontal Inflammation with No Apparent Sex Differences

**Figure 3:**
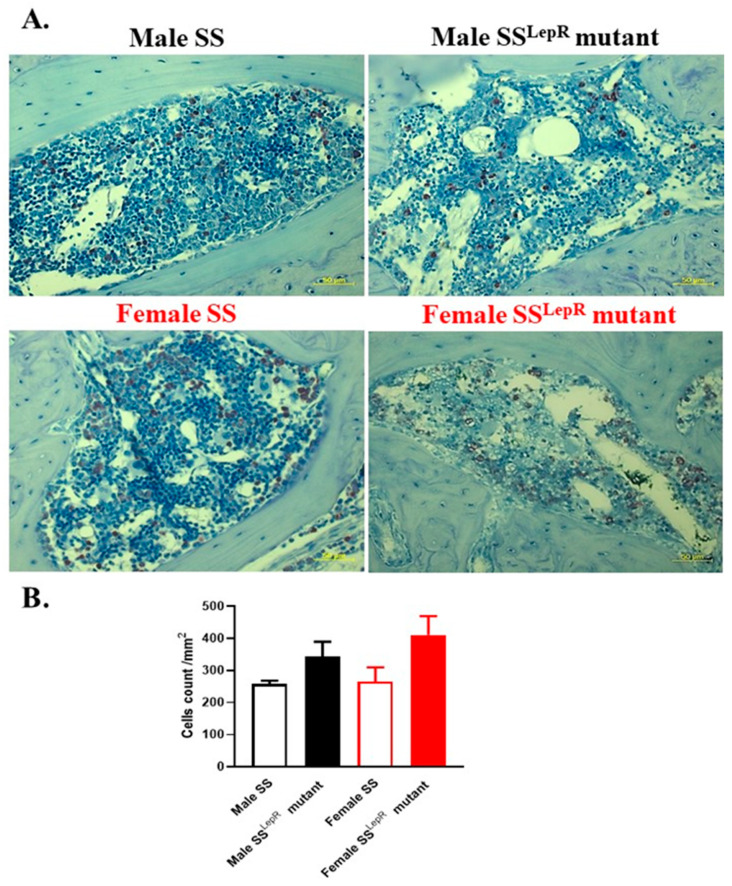
Obesity did not significantly increase periodontium myeloperoxidase staining in both sexes. Representative images of immune–histochemical staining of myeloperoxidase (**A**) at 200× magnification and average number of myeloperoxidase positive cells per mm^2^ (**B**) in maxilla sections from both male and female lean Dahl salt-sensitive (SS) and obese SS leptin receptor mutant (SS^LepR^ mutant) rats. Values are presented as mean ± SEM (n = 4/group).

**Figure 4:**
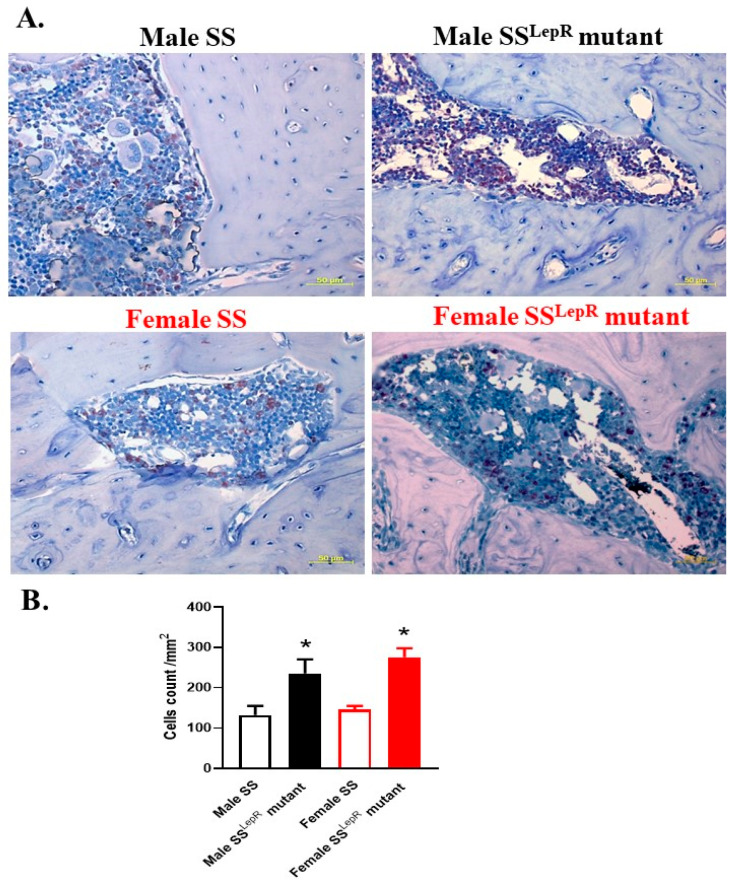
Obesity increased periodontium IL-6 staining in both sexes. Representative images of immune–histochemical staining of IL-6 (**A**) at 200× magnification and average number of IL-6-positive cells per mm^2^ (**B**) in maxilla sections from both male and female lean Dahl salt-sensitive (SS) and obese SS leptin receptor mutant (SS^LepR^ mutant) rats. Values are presented as mean ± SEM. * *p* < 0.05 vs. male or female lean Dahl SS rats (n = 4/group).

**Figure 5:**
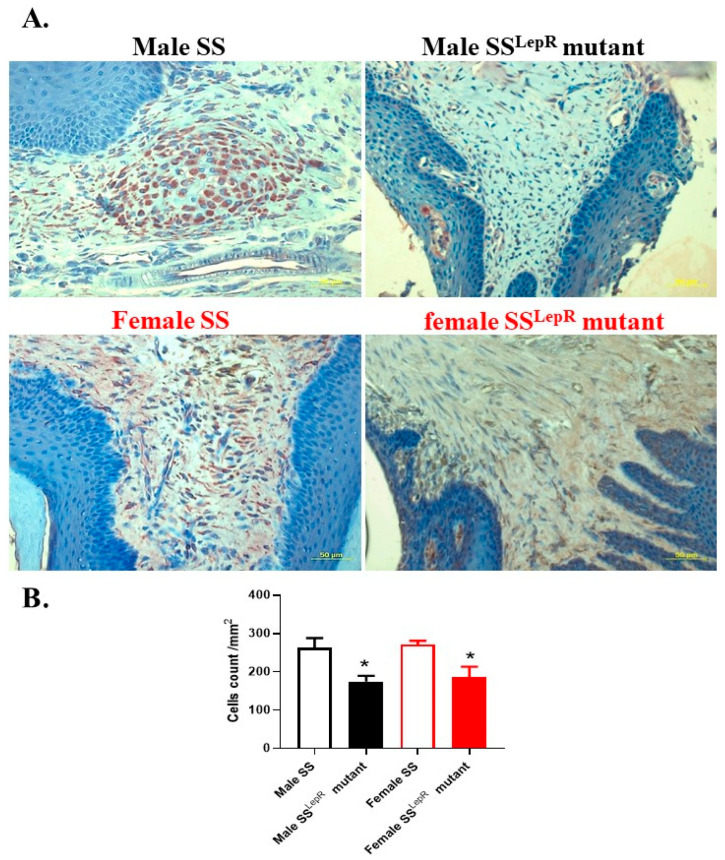
Obesity decreased periodontium IL-10 staining in both sexes. Representative images of immune–histochemical staining of IL-10 (**A**) at 200× magnification and average number of IL-10-positive cells per mm^2^ (**B**) in maxilla sections from both male and female lean Dahl salt-sensitive (SS) and obese SS leptin receptor mutant (SS^LepR^ mutant) rats. Values are presented as mean ± SEM. * *p* < 0.05 vs. male or female lean Dahl SS rats (n = 4/group).

Using immune–histochemical staining, periodontal myeloperoxidase expression levels, a marker of inflammation and the most abundant protein in neutrophils that is known to participate in the initiation and progression of periodontal disease [24], were evaluated (Figure 3). Representative images of myeloperoxidase-positive cells in hematopoietic marrow (brown staining) in the periodontium were detected (Figure 3A). Although myeloperoxidase-positive cells appeared to be higher in obese vs. lean rats, this elevation was not significant, with no apparent sex differences (Figure 3B). Immuno-histochemical assessments of the inflammatory cytokine IL-6, which is known to stimulate osteoclast formation leading to bone resorption [25], were also performed in hematopoetic bone marrow in both rat strains (Figure 4A). The periodontium IL-6-positive cells were significantly increased in obese male and female SS^LepR^ mutant rats relative to their lean SS controls, with no apparent sex difference (Figure 4B). The elevation in the level of inflammatory IL-6 in the periodontium of obese SS^LepR^ mutant rats vs. SS rats was associated with a significant decrease in anti-inflammatory IL-10 staining (Figure 5A). IL-10 staining significantly decreased in obese SS^LepR^ mutant rats vs. lean SS rats in both sexes, with no apparent sex difference (Figure 5B).

## 4. Discussion

Periodontal disease and obesity are major public health problems characterized by chronic low-grade inflammation [26], and obesity is known to exacerbate periodontal inflammation and injury [16,17]. Sex differences were noted in the incidence and progression of periodontal inflammation, whereby obese males are at a significantly higher risk of severe periodontitis than obese females [4,5]. However, the molecular and cellular mechanisms of these sex differences remain unclear. In the current study, obese SS^LepR^ mutant rats vs. lean SS rats were used to address the role of inflammation in the sex differences in periodontitis. Male and female obese SS^LepR^ mutant rats showed significant elevations in markers of systemic oxidative stress and inflammation relative to lean SS rats with no apparent sex differences. The elevation in systemic oxidative stress and inflammation in obese SS^LepR^ mutant rats did not translate to alveolar bone loss; however, obese SS^LepR^ mutant rats showed significant elevations in the inflammatory IL-6 and reductions in the anti-inflammatory IL-10 staining in the periodontium relative to the lean SS rats, with no apparent sex differences. These data suggest that obesity-induced elevations in systemic and periodontal inflammation are not enough to induce alveolar bone loss in obese SS^LepR^ mutant rats relative to their lean SS counterparts.

Obesity is a growing pandemic, and is associated with vascular injury, predisposing patients to cardio-metabolic syndrome such as type II diabetes, hyperlipidemia, hypertension, and insulin-resistant syndrome. Periodontitis is an inflammatory disease that causes the destruction of the tooth-supporting structures, including the bone. Oxidative stress, pro-inflammatory cytokines, and chemokines play a role not only in the incidence but also in the progression of periodontitis. Obesity is known to exacerbate the progression of periodontal inflammation, and this could be attributed to the elevation in oxidative stress and inflammatory cytokine levels in obese subjects. Clinically, studies have shown sex differences in vascular function and periodontal disease, whereby males have higher vascular dysfunctions and prevalences of periodontal disease (56%) compared to age-matched females (38%) [4,5]. Increases in weight are positively associated with vascular injury and periodontitis in both sexes [10,11,27]; however, there is controversy in the literature regarding the relative susceptibility of males vs. females to obesity-induced vascular injury. Clinical, population, and basic science studies suggest the enhanced susceptibility of females to obesity-induced vascular dysfunction [28,29], whereas other studies indicate females maintain cardiovascular protection during obesity [30,31,32]. In a mice model of periodontitis, one study has shown conflicting data to the clinical findings, whereby female mice displayed significantly increased periodontal bone loss, with elevations in pro-inflammatory cytokines’ expression and higher numbers of oral bacteria than males [6]. Thus, more work is needed to better understand the impact of sex on the obesity-induced exacerbation of periodontal inflammation.

The obese SS^LepR^ mutant rat model was created on the Dahl SS genetic background using Zinc-finger technology [16]. Zinc-finger constructs targeting a specific region in exon 11 of the leptin receptor gene on chromosome 5 were employed to induce a frameshift deletion, resulting in the creation of the SS^LepR^ mutant strain. The SS^LepR^ mutant strain is visually obese and develops dyslipidemia as early as 6 weeks of age [17]. Although insulin resistance and hyperinsulinemia were observed in the SS^LepR^ mutant strain by 6 weeks of age, the mice do not develop hyperglycemia until 18 weeks of age [16,17]. The SS^LepR^ mutant strain also develops severe hypertension after 14 weeks of age, whereas progressive proteinuria is observed early, at 6 weeks of age, before elevations in arterial pressure, and is independent of hyperglycemia [16,17]. In the current study, 11–12-week-old male and female obese SS^LepR^ mutant and lean SS rats were utilized to determine if obesity exacerbates periodontal inflammation and assess possible sex differences in periodontal inflammation independent of hyperglycemia and hypertension. Consistent with the previously published data [16,17], body weight, cholesterol and insulin levels were elevated in the obese SS^LepR^ mutant strain relative to the lean SS rat strain. A sex difference in body weight was noted in both lean SS and obese SS^LepR^ mutant rats, whereby males had higher body weight than females in both strains. Hemoglobin A1c significantly increased in obese male SS^LepR^ mutant rats compared with lean SS rats, and this elevation was not observed in female obese SS^LepR^ mutant rats, suggesting that obese male SS^LepR^ mutant rats might be more sensitive to obesity-induced cardio-metabolic symptoms than females. However, there were no differences in blood glucose levels in both lean SS and obese SS^LepR^ mutant rat strains, making our obese rat model ideal to address the effects of obesity on periodontal inflammation independent of hyperglycemia or hypertension as confounding factors for the progression of vascular inflammation.

Previous findings suggest that obesity also increases the incidence of periodontal disease [33], whereby elevations in oxidative stress and inflammatory cytokines production are key elements in the progression of vascular injury [9,34]. Periodontitis could also affect systemic health and increase the risk of cardiovascular disease [3,35,36]. Obese SS^LepR^ mutant rats showed elevations in plasma TBARs and MCP-1 levels as markers of oxidative stress and inflammation, respectively, relative to their lean control SS rats, with no apparent sex differences in the current study. These data are inconsistent with the previous findings, wherein males displayed higher levels of oxidative stress and inflammation than females [37,38]. However, the elevation in systemic oxidative stress and inflammation did not translate to significant pathophysiological changes in periodontium or alveolar bone loss. Although cross-sections of periodontium interseptal bone showed the presence of osteoclasts in obese SS^LepR^ mutant rats vs. lean SS, there was no evidence of alveolar bone loss in obese SS^LepR^ mutant rats, suggesting that elevations in systemic oxidative stress and inflammation in obese SS^LepR^ mutant rats might not be enough to drive significant pathophysiological changes in periodontium nor alveolar bone loss.

Sex differences in periodontal inflammation in obese SS^LepR^ mutant rats and lean SS rats were also examined. Previous findings suggest that an elevation in salivary myeloperoxidase correlates with increased body mass index in obese subjects as a result of chronic inflammation in the gingiva [39]. Thus, myeloperoxidase staining in maxilla sections isolated from both rat strains was evaluated, since myeloperoxidase is an established marker of inflammation and the most abundant protein in neutrophils that is known to participate in the initiation and progression of periodontal disease [24]. Although the number of myeloperoxidase-positive cells in the periodontium of obese SS^LepR^ mutant rats tended to be higher than in lean SS rats in both sexes, these changes were not significant, and there was no sex difference in myeloperoxidase staining in both rat strains. These data suggest that neither obesity nor the elevation in systemic oxidative stress and/or inflammation is enough to drive an increased neutrophilic production of myeloperoxidase that will initiate the progression of periodontal inflammation.

Studies suggest that dental plaque bacteria are the main causative factor for periodontal disease, acting via increasing the production of inflammatory cytokines that will aggravate tissue damage. IL-6 is known to play a role in pathogenesis or periodontitis via the induction of osteoclast differentiation to facilitate bone resorption as well as the inhibition of bone formation [40,41]. Obesity is known to activate inflammatory responses with increased production in pro-inflammatory IL-6 in the periodontium, triggering periodontal inflammation [42]. In the current study, the numbers of periodontium IL-6-positive cells in maxilla sections from obese male and female SS^LepR^ mutant rats were significantly elevated when compared to their lean SS rats, with no apparent sex differences. On the other hand, IL-10 is an anti-inflammatory cytokine that functions to reduce the severity of inflammatory response in patients with periodontal disease [43]. Thus, IL-10 could play a crucial role in limiting the duration and extent of inflammatory responses in periodontal disease patients [43]. A previous study showed a positive association between IL-6 and gingival index, but a negative association between IL-10 levels and gingival index as a periodontal parameter in obese subjects, since salivary IL-6 increased and IL-10 decreased in obese patients with gingivitis when compared to non-obese subjects with gingivitis [44]. In our study, the numbers of periodontium IL-10-positive cells in maxilla sections from obese males and females significantly decreased when compared to lean SS controls, with no apparent sex differences. Contrary to clinical studies [4,5], the absence of an apparent gender difference in periodontal inflammation in our obese rat model could be attributed to the absence of predisposing factors such as smoking and poor dental hygiene.

## 5. Conclusions

In summary, obesity increases systemic and periodontal inflammation; however, these changes are not enough to induce periodontitis and alveolar bone loss in the obese SS^LepR^ mutant rat model. Although a sex difference is observed in terms of body weight in both lean SS and obese SS^LepR^ mutant rats, whereby males have higher body weights than females, there are no apparent sex differences in systemic or periodontal inflammation in obese SS^LepR^ mutant rats. In conclusion, obesity is not enough, at least in the obese SS^LepR^ mutant rat model, to induce periodontitis.

## 6. Future Direction

Since periodontitis is primarily an infectious–inflammatory disease triggered by the accumulation of bacterial biofilm, future studies should focus on experimentally induced periodontitis in male and female lean SS and obese SS^LepR^ mutant rats to determine if obesity will trigger periodontal inflammation and bone loss in both sexes, and to determine possible sex differences in the obesity-induced exacerbation of periodontal inflammation in an obese SS^LepR^ mutant rat model.

## Figures and Tables

**Table 1:** Sex differences were observed in obesity-induced increases in body weight and plasma hemoglobin A1c levels. Metabolic parameters in male and female lean Dahl salt-sensitive (SS) and obese SS leptin receptor mutant (SS^LepR^ mutant) rats at 11–12 weeks of age. Values are presented as means ± SEM. * *p* < 0.05 vs. SS rats within the same sex and ^#^ *p* < 0.05 vs. male rats within the same strain (n = 6–7/group).

Parameter	Male SS	Male SS^LepR^ Mutant	Female SS	Female SS^LepR^ Mutant
Body weight (gm)	314 ± 17	401 ± 26 *	210 ± 7 ^#^	321 ± 17 *^#^
HbA1c %	4.5 ± 0.1	5.6 ± 0.1 *	4.3 ± 0.2	4.8 ± 0.1 ^#^
Glucose (mg/dL)	102 ± 5.1	101 ± 9.4	101 ± 4.6	98 ± 5.6
Cholesterol (mg/dL)	98 ± 0.3	195 ± 0.7 *	97 ± 0.4	198 ± 1.3 *
Insulin (µIU/ml)	11.3 ± 1.9	47.9 ± 12.4 *	7.9 ± 1.7 ^#^	49.8 ± 12.8 *

## Data Availability

The data presented in this study will be available on request from the corresponding author.
